# Supplementary material for: Reliability of AlphaFold2 Models in Virtual Drug Screening: A Focus on Selected Class A GPCRs
Source: Int J Mol Sci. 2024 Sep 21;25(18):10139. doi: 10.3390/ijms251810139 (PMC11432040; doi:10.3390/ijms251810139)
Supplement: Supplementary file 1 [file ijms-25-10139-s001.zip › ijms-3206836-supplementary.pdf]

Supplementary Materials

Table S1. The Activity of selected ligands was downloaded from the Zinc20 Database.

Zinc ID	Receptor	pKi
**ZINC000013826578**	D (2) dopamine receptor	7.33
	5-hydroxytryptamine receptor 1A	6.30
**ZINC000045347746**	Delta-type opioid receptor	10.00
	Kappa-type opioid receptor	10.82
	Mu-type opioid receptor	9.47
	Nociceptin receptor	6.87
**ZINC000073138555**	Substance-P receptor	10.30
	Neuromedin-K receptor	8.80
**ZINC000027193871**	Neuropeptide Y receptor type 1	9.12
**ZINC000653730896**	D (2) dopamine receptor	7.30
	D (3) dopamine receptor	8.90
	Muscarinic acetylcholine receptor M1	6.96
	Muscarinic acetylcholine receptor M3	6.61
**ZINC000036178926**	Lysophosphatidic acid receptor 1	6.81
	Lysophosphatidic acid receptor 3	6.03
**ZINC000013826577**	D (2) dopamine receptor	8.85
	5-hydroxytryptamine receptor 1A	6.98
**ZINC000653730899**	D (2) dopamine receptor	7.30
	D (3) dopamine receptor	8.90
	Muscarinic acetylcholine receptor M1	6.96
	Muscarinic acetylcholine receptor M3	6.61
**ZINC000036178877**	Lysophosphatidic acid receptor 1	7.74
	Lysophosphatidic acid receptor 3	6.76
**ZINC000013826580**	D (2) dopamine receptor	9.52
	5-hydroxytryptamine receptor 1A	7.30
**ZINC000653730898**	D (2) dopamine receptor	7.30
	D (3) dopamine receptor	8.90
	Muscarinic acetylcholine receptor M1	6.96
	Muscarinic acetylcholine receptor M3	6.61
**ZINC000653730897**	D (2) dopamine receptor	7.30
	D (3) dopamine receptor	8.90
	Muscarinic acetylcholine receptor M1	6.96
	Muscarinic acetylcholine receptor M3	6.61
**ZINC000013757083**	5-hydroxytryptamine receptor 2A	9.52
	5-hydroxytryptamine receptor 2B	9.59
	5-hydroxytryptamine receptor 2C	8.94
	5-hydroxytryptamine receptor 1A	8.37
**ZINC000043203224**	Sphingosine 1-phosphate receptor 1	8.52
	Sphingosine 1-phosphate receptor 3	7.21
	Sphingosine 1-phosphate receptor 5	6.94
**ZINC000013982068**	Adenosine receptor A1	9.40
	Adenosine receptor A2a	10.00
**ZINC000053276566**	C-C chemokine receptor type 2	8.80
**ZINC000035322879**	C-C chemokine receptor type 2	8.90
	C-C chemokine receptor type 4	6.10
	C-C chemokine receptor type 5	6.60
**ZINC000045347746**	Delta-type opioid receptor	10.00
	Kappa-type opioid receptor	10.82

	Mu-type opioid receptor	9.47
	Nociceptin receptor	6.87
**ZINC000045392798**	Delta-type opioid receptor	8.60
	Kappa-type opioid receptor	9.47
	Mu-type opioid receptor	9.33
	Nociceptin receptor	8.38
**ZINC000043207236**	Orexin receptor type 1	9.93
	Orexin receptor type 2	10.11
**ZINC000064560440**	Neuropeptide Y receptor type 2	8.70
**ZINC000022064449**	D (2) dopamine receptor	8.20
	D (3) dopamine receptor	9.68
**ZINC000022064446**	D (2) dopamine receptor	9.47
	D (3) dopamine receptor	9.70
**ZINC000013604966**	5-hydroxytryptamine receptor 1A	8.80
	5-hydroxytryptamine receptor 1B	10.00
	5-hydroxytryptamine receptor 1D	9.94
**ZINC000013604972**	5-hydroxytryptamine receptor 1A	8.02
	5-hydroxytryptamine receptor 1B	9.96
	5-hydroxytryptamine receptor 1D	9.80
**ZINC000100015780**	Muscarinic acetylcholine receptor M1	9.36
	Muscarinic acetylcholine receptor M2	9.38
	Muscarinic acetylcholine receptor M3	9.72
	Muscarinic acetylcholine receptor M4	9.42
	Muscarinic acetylcholine receptor M5	8.98
**ZINC000071317350**	Melatonin receptor type 1A	8.85
	Melatonin receptor type 1B	9.22
**ZINC000022582179**	Alpha-2A adrenergic receptor	10.00
	Alpha-2C adrenergic receptor	10.00
**ZINC000006467610**	Type-1 angiotensin II receptor	9.40
	Type-2 angiotensin II receptor	9.40
**ZINC000034984449**	Muscarinic acetylcholine receptor M2	8.80
	Muscarinic acetylcholine receptor M3	9.60
**ZINC000034984672**	Muscarinic acetylcholine receptor M2	10.25
	Muscarinic acetylcholine receptor M3	10.31
**ZINC000034984565**	Muscarinic acetylcholine receptor M2	8.59
	Muscarinic acetylcholine receptor M3	9.57
**ZINC000034872896**	Muscarinic acetylcholine receptor M3	9.74
**ZINC000034984673**	Muscarinic acetylcholine receptor M1	9.94
	Muscarinic acetylcholine receptor M2	9.86
	Muscarinic acetylcholine receptor M3	9.89
	Muscarinic acetylcholine receptor M4	9.94
	Muscarinic acetylcholine receptor M5	9.77
**ZINC000043152664**	Muscarinic acetylcholine receptor M3	9.74
**ZINC000034984802**	Muscarinic acetylcholine receptor M3	9.66
**ZINC000034984677**	Muscarinic acetylcholine receptor M3	9.79
**ZINC000073138544**	Substance-P receptor	10.10
	Neuromedin-K receptor	8.30
**ZINC000073138552**	Substance-P receptor	10.30
	Neuromedin-K receptor	8.80
**ZINC000073141571**	Substance-P receptor	10.20
	Neuromedin-K receptor	9.00
**ZINC000082138033**	Substance-P receptor	10.10
	Neuromedin-K receptor	8.30
**ZINC000028570279**	Cannabinoid receptor 1	10.00
**ZINC000084670829**	Cannabinoid receptor 1	9.96
**ZINC000084653533**	Cannabinoid receptor 1	9.96

**ZINC000084653534**	Cannabinoid receptor 1	9.96
**ZINC000022933256**	Alpha-2A adrenergic receptor	8.89
	Alpha-2C adrenergic receptor	10.00
	Alpha-2B adrenergic receptor	8.35
**ZINC000038408115**	Muscarinic acetylcholine receptor M2	9.77
	Histamine H3 receptor	7.74
**ZINC000095552079**	Adenosine receptor A1	9.30
	Adenosine receptor A2a	10.00
**ZINC000084403338**	Adenosine receptor A2a	10.00
**ZINC000003783617**	Delta-type opioid receptor	9.51
	Kappa-type opioid receptor	9.49
	Mu-type opioid receptor	10.68
**ZINC000026392975**	Muscarinic acetylcholine receptor M2	9.85
**ZINC000026387032**	Muscarinic acetylcholine receptor M2	9.54
**ZINC000026394331**	Muscarinic acetylcholine receptor M2	9.70
**ZINC000026649493**	Neuropeptide Y receptor type 1	9.12
**ZINC000028136015**	Adenosine receptor A1	8.96
	Adenosine receptor A2a	10.00
**ZINC000003951093**	Neuromedin-B receptor	9.77
	Gastrin-releasing peptide receptor	9.00
**ZINC000003940643**	Delta-type opioid receptor	7.53
	Kappa-type opioid receptor	10.70
	Mu-type opioid receptor	9.02
**ZINC000028569846**	Cannabinoid receptor 1	10.00
**ZINC000022931162**	Muscarinic acetylcholine receptor M1	5.22
	Muscarinic acetylcholine receptor M2	5.32
	C-C chemokine receptor type 5	8.96
**ZINC000028564410**	5-hydroxytryptamine receptor 1A	9.52
	5-hydroxytryptamine receptor 1B	10.00
**ZINC000003925861**	Proteinase-activated receptor 1	8.96
**ZINC000100009278**	Muscarinic acetylcholine receptor M1	9.60
	Muscarinic acetylcholine receptor M2	9.46
	Muscarinic acetylcholine receptor M3	9.92
	Muscarinic acetylcholine receptor M4	9.98
	Muscarinic acetylcholine receptor M5	9.68
**ZINC000000537822**	D (1A) dopamine receptor	8.21
	D (2) dopamine receptor	10.12
	D (3) dopamine receptor	10.22
	D (4) dopamine receptor	9.13
**ZINC000002568244**	Cannabinoid receptor 1	9.89
	Cannabinoid receptor 2	9.82
	N-arachidonoyl glycine receptor	5.22
	G-protein coupled receptor 55	5.79
**ZINC000043078698**	Prostaglandin E2 receptor EP3 subtype	8.90
**ZINC000003802871**	Delta-type opioid receptor	10.52
	Kappa-type opioid receptor	8.91
	Mu-type opioid receptor	9.44
	Nociceptin receptor	10.52
**ZINC000003995590**	Delta-type opioid receptor	9.52
	Kappa-type opioid receptor	10.57
	Mu-type opioid receptor	9.66
**ZINC000003820128**	Cannabinoid receptor 1	10.00
**ZINC000003820123**	Cannabinoid receptor 1	10.00
**ZINC000045347746**	Delta-type opioid receptor	10.00
	Kappa-type opioid receptor	10.82
	Mu-type opioid receptor	9.47

	Nociceptin receptor	6.87
**ZINC000004424097**	Gonadotropin-releasing hormone receptor	9.70
**ZINC000000598040**	5-hydroxytryptamine receptor 2A	10.00
	5-hydroxytryptamine receptor 2C	7.89
	D (2) dopamine receptor	5.89
	Histamine H1 receptor	6.00
**ZINC000026384753**	Muscarinic acetylcholine receptor M2	9.64
**ZINC000003979278**	Muscarinic acetylcholine receptor M2	11.00
**ZINC000026479542**	C-C chemokine receptor type 5	8.85
**ZINC000022930620**	Alpha-2A adrenergic receptor	9.52
	Alpha-2C adrenergic receptor	10.00
	Alpha-2B adrenergic receptor	6.75

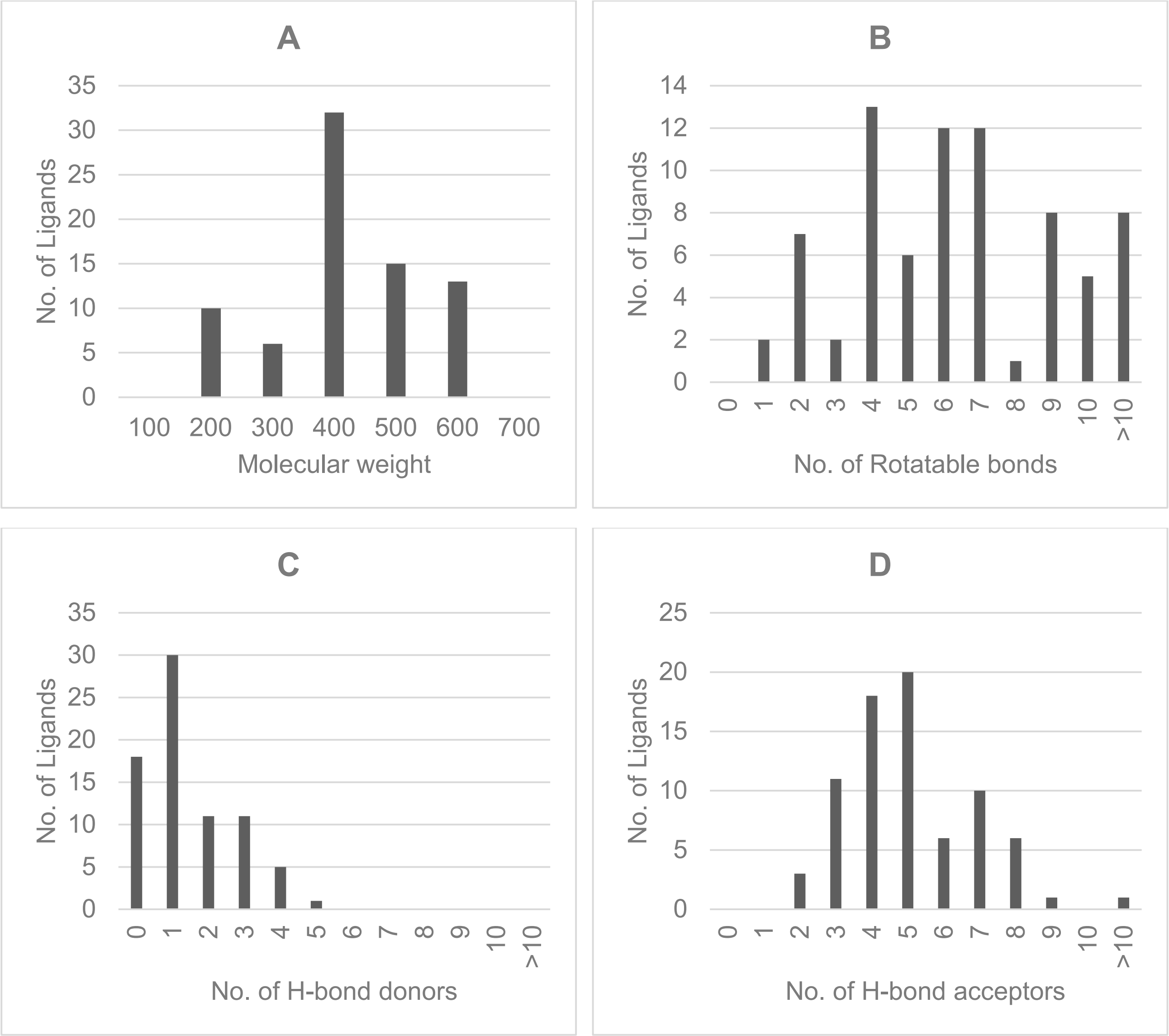

Figure S1. Histograms of distributions of selected physiochemical properties for the 76 active ligands included in the docking set. **A)** Ligand molecular weight (Median: 462.5, Mean: 469.7, Standard deviation (SD): 114.8). **B)** The number of rotatable bonds of ligands (Median: 6.0, Mean: 6.7, SD: 4.0). **C)** The number of H-bond donors of ligands (Median: 1.0, Mean: 1.4, SD: 1.3). **D)** The number of H-bond acceptors of ligands (Median: 5.0, Mean: 5.1, SD: 1.8).

Table S2. Features of the X-ray structures downloaded from the Protein Data Bank.

Receptor subfamily	Protein Name	IUPHAR	X-ray PDB	Resolution (Å)	Crystal Ligand	Cryo-EM PDB	Resolution (Å)	AlphaFold2 Code
**5-Hydroxytryptamine**	5-hydroxytryptamine receptor 2B	5-HT2B	4IB4	2.7	ergotamine	7SRQ	2.7	AF-P41595-F1
**5-Hydroxytryptamine**	5-hydroxytryptamine receptor 2C	5-HT2C	6BQH	2.7	ritanserin	8DPF	2.8	AF-P28335-F1
**5-Hydroxytryptamine**	5-hydroxytryptamine receptor 5A	5-HT5A	7UM4	2.8	CHEMBL3654198	7UM7	2.8	AF-P47898-F1
**Angiotensin**	Type-1 angiotensin II receptor	AT1	4ZUD	2.8	OLMESARTAN	7F6G	2.9	AF-P30556-F1
**Bombesin**	Gastrin-releasing peptide receptor	BB2	7W41	3	PD 176252	7W40 or 7W3Z	3.0	AF-P30550-F1
**Cannabinoid**	Cannabinoid receptor 1	CB1	5U09	2.6	taranabant	8GHV	2.8	AF-P21554-F1
**Cannabinoid**	Cannabinoid receptor 2	CB2	5ZTY	2.8	AM10257	8GUR	2.8	AF-P34972-F1
**Cholecystokinin**	Cholecystokinin receptor type A	CCK1	7F8Y	2.5	DEVAZEPIDE	7MBX	2.0	AF-P32238-F1
**Leukotriene**	Leukotriene B4 receptor 1	BLT1	7K15	2.9	N-(tert-butylsulfonyl)-4-fluoro-2-{(3S,4R)-4-hydroxy-3-[(pyridin-2-yl)methyl]-3,4-dihydro-2H-1-benzopyran-7-yl}benzamide	7VKT	2.9	AF-Q15722-F1
**Chemokine**	C-C chemokine receptor type 2	CCR2	6GPX	2.7	MK-0812	7XA3	2.9	AF-P41597-F1
**Chemokine**	C-C chemokine receptor type 5	CCR5	4MBS	2.7	maraviroc	7F1S	2.8	AF-P51681-F1
**Dopamine**	D(2) dopamine receptor	D2	6CM4	2.9	risperidone	7JVR	2.8	AF-P14416-F1
**Dopamine**	D(3) dopamine receptor	D3	3PBL	2.9	eticlopride	8IRT or 7CMV	2.7	AF-P35462-F1
**Endothelin**	Endothelin receptor type B	ETB	5X93	2.2	K-8794	8IY5	2.8	AF-P24530-F1
**Free fatty acid**	Free fatty acid receptor 1	FFA1	5TZR	2.2	MK-8666	8EIT	2.8	AF-O14842-F1
**Ghrelin**	Growth hormone secretagogue receptor type 1	ghrelin	7F83	2.9	PF-05190457	7NA7 or 7NA8	2.7	AF-Q92847-F1
**A orphans**	G-protein coupled receptor 52	GPR52	6LI0	2.2	derivative 17 [Nakahataet al., 2018]	8HMP	2.8	AF-Q9Y2T5-F1
**Lysophospholipid (LPA)**	Lysophosphatidic acid receptor 1	LPA1	4Z35	2.9	ONO-9910539	7TD0	2.8	AF-Q92633-F1
**Acetylcholine (muscarinic)**	Muscarinic acetylcholine receptor M3	M3	4U15	2.8	tiotropium	8EA0	2.8	AF-P08483-F1
**Acetylcholine (muscarinic)**	Muscarinic acetylcholine receptor M4	M4	5DSG	2.6	tiotropium	7TRP	2.4	AF-P08173-F1
**Tachykinin**	Substance-P receptor	NK1	6HLP	2.2	netupitant	8U26	2.5	AF-P25103-F1
**Orexin**	Orexin receptor type 2	OX2	5WQC	2	EMPA	7L1V	3.0	AF-O43614-F1
**P2Y**	P2Y purinoceptor 1	P2Y1	4XNW	2.7	MRS2500	7XXH	2.9	AF-P47900-F1
**P2Y**	P2Y purinoceptor 12	P2Y12	4PXZ	2.5	2MeSADP	7XXI	3.0	AF-Q9H244-F1
**Lysophospholipid (S1P)**	Sphingosine 1-phosphate receptor 1	S1P1	3V2Y	2.8	W146	7TD4	2.6	AF-P21453-F1
**Lysophospholipid (S1P)**	Sphingosine 1-phosphate receptor 5	S1P5	7YXA	2.2	4-[6-(2-naphthalen-1-ylethoxy)-2,3,4,5-tetrahydro-1H-3-	7T10	2.5	AF-Q9H228-F1

					benzazepin-3-ium-3-yl]butanoic acid			
**Somatostatin**	Somatostatin receptor type 2	SST2	7XN9	2.6	L-054,522	7YON	3.0	AF-P30874-F1
**Neuropeptide Y**	Neuropeptide Y receptor type 2	Y2	7DDZ	2.8	CHEMBL1823578	7EJ8	3.0	AF-P49146-F1
**Adrenoceptors**	Alpha-2A adrenergic receptor	α2A	6KUX	2.7	RSC	8DCS	2.5	AF-P08913-F1
**Adrenoceptors**	Beta-1 adrenergic receptor	β1	4BVN	2.1	(S)-Cyanopindolol	8F7S	3.0	AF-P07700-F1
**Opioid**	Delta-type opioid receptor	δ	4N6H	1.8	naltrindole	8FEG	2.5	AF-P41143-F1
**Opioid**	Kappa-type opioid receptor	κ	4DJH	2.9	JDTic	7T2G	2.5	AF-P41145-F1
**Opioid**	Mu-type opioid receptor	μ	5C1M	2.1	BU72	7SRQ	2.7	AF-P42866-F1

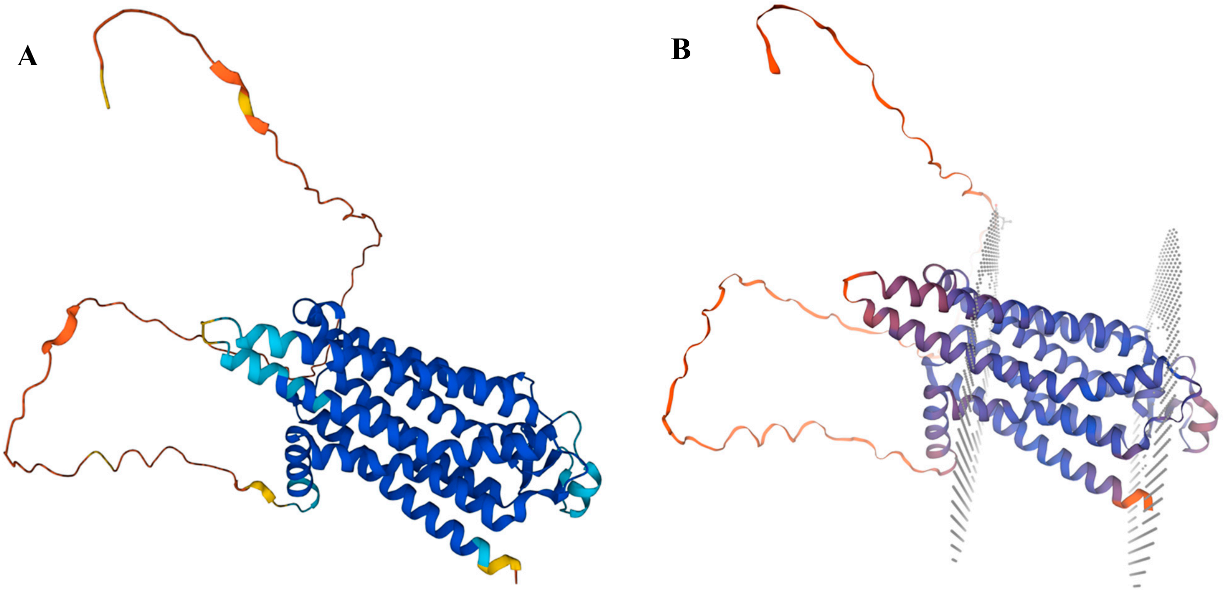

Figure S2. The Quality of AF2 model of Adenosine receptor A2a. **A, pLDDT:** blue color indicates very high model confidence (pLDDT > 90); cyan color indicates high model confidence (pLDDT > 70); yellow color indicates low model confidence (pLDDT > 50); orange color indicates very low model confidence (pLDDT < 50). **B, QMEANDisCo:** the darker blue color indicates higher model confidence, and the darker orange color indicates lower model confidence.

Table S3. 2D Ligand Interaction of native ligand of the X-ray structure and the top-scored ligand identified using AF2 models.

5-HT2C X-ray Structure		Corresponding AF2 model	
Crystal Ligand	PLP-Fitness	Ligand Code	PLP-Fitness
ritanserin	98.26	ZINC000036178926	113.514
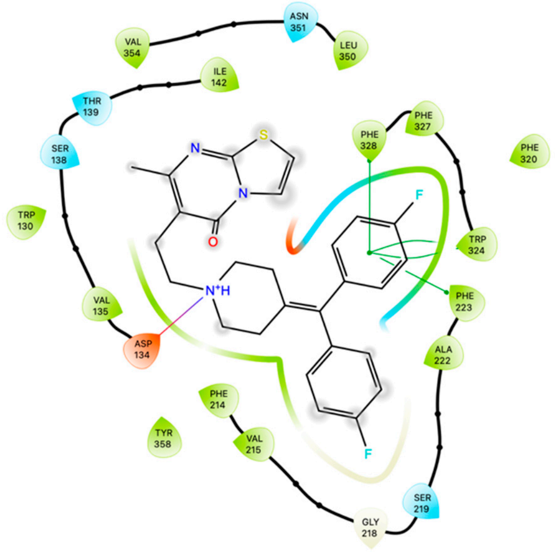		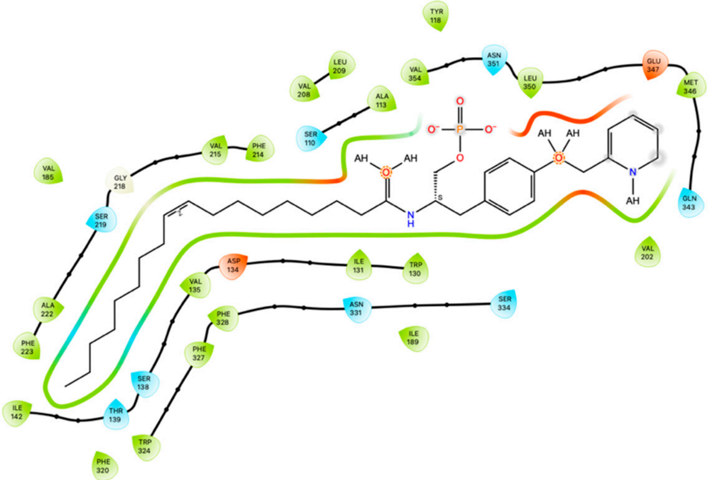	
5-HT5A X-ray Structure		Corresponding AF2 model	
Crystal Ligand	PLP-Fitness	Ligand Code	PLP-Fitness
CHEMBL3654198	82.43	ZINC000036178877	88.282
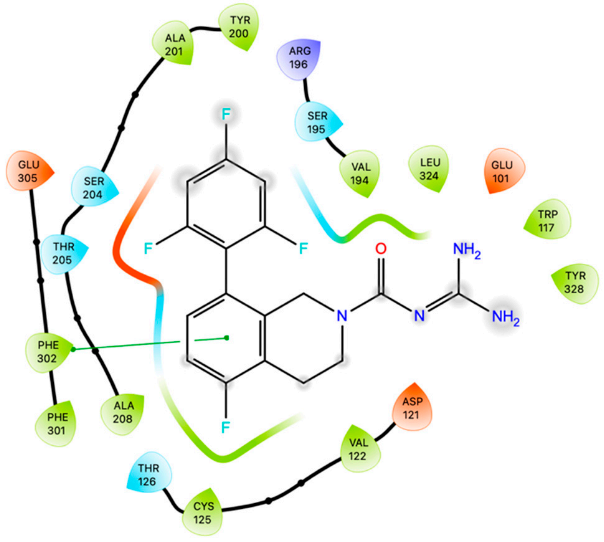		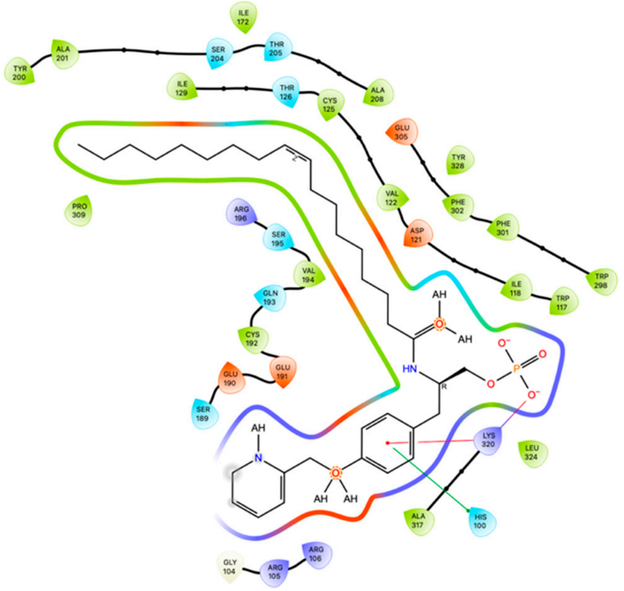	

AT1 X-ray Structure		Corresponding AF2 model	
Crystal Ligand	PLP-Fitness	Ligand Code	PLP-Fitness
OLMESARTAN	80.74	ZINC000036178877	106.787

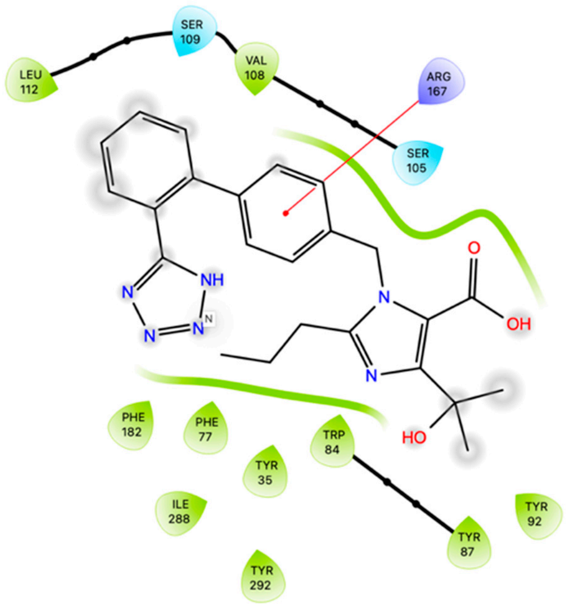

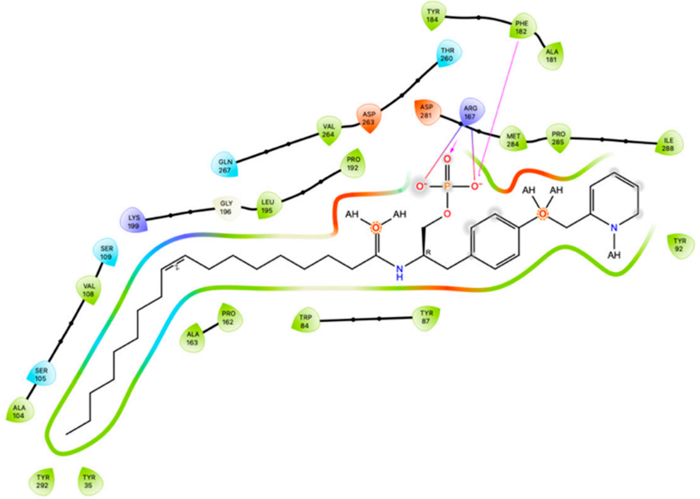

BB2 X-ray Structure		Corresponding AF2 model	
Crystal Ligand	PLP-Fitness	Ligand Code	PLP-Fitness
PD 176252	95.70	ZINC000036178926	103.382

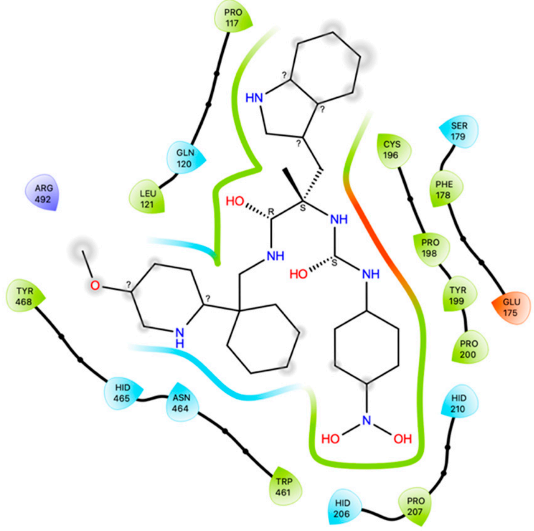

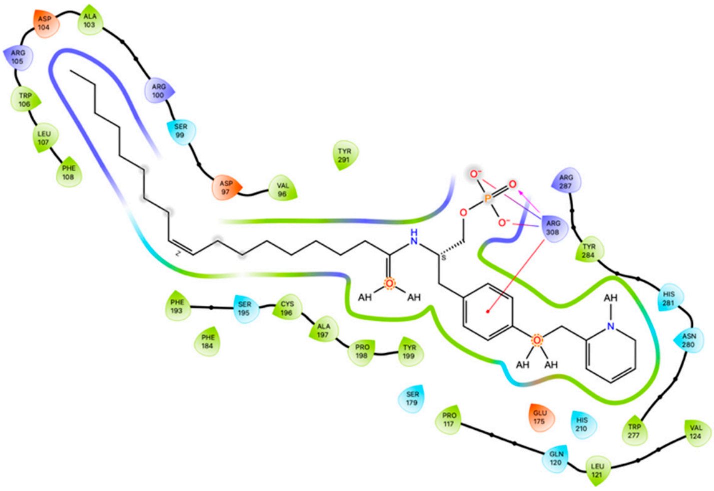

CB1 X-ray Structure		Corresponding AF2 model	
Crystal Ligand	PLP-Fitness	Ligand Code	PLP-Fitness
taranabant	101.14	ZINC000027193871	103.143
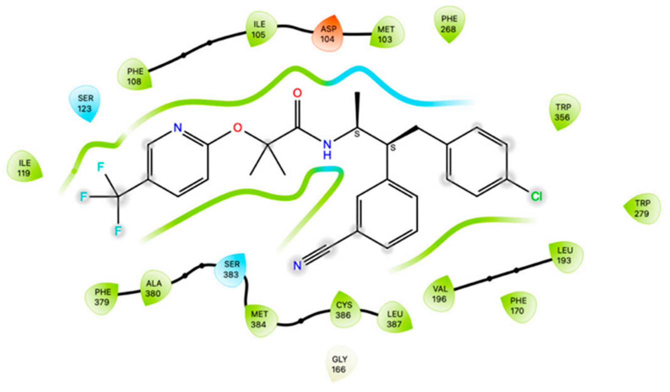		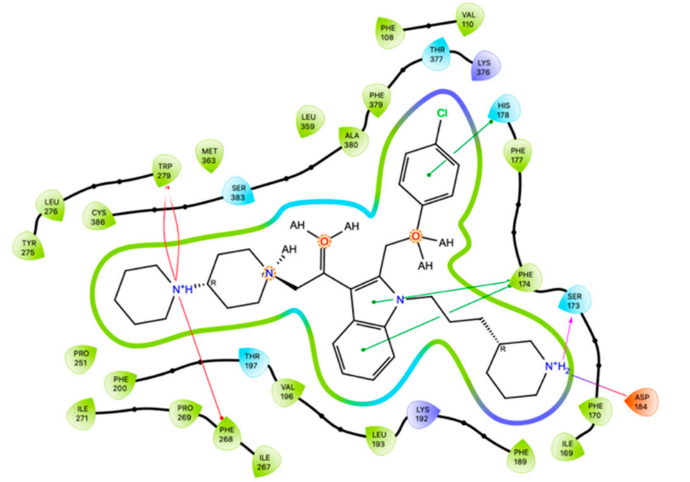	

CB2 X-ray Structure		Corresponding AF2 model	
Crystal Ligand	PLP-Fitness	Ligand Code	PLP-Fitness
AM10257	103.26	ZINC000027193871	122.306
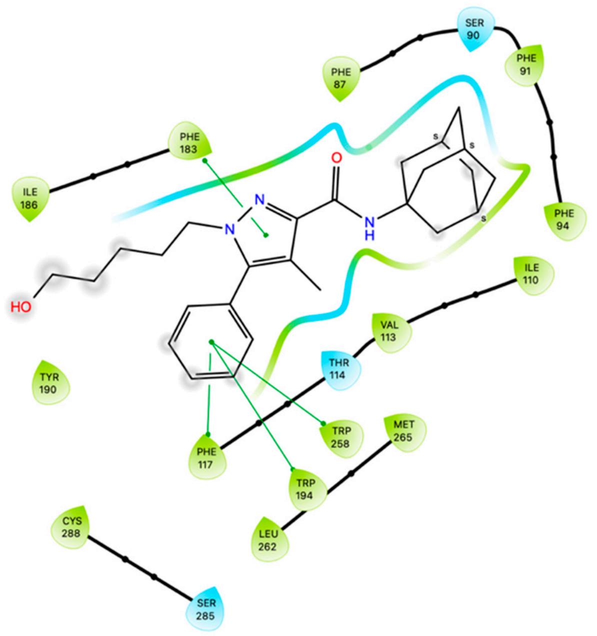		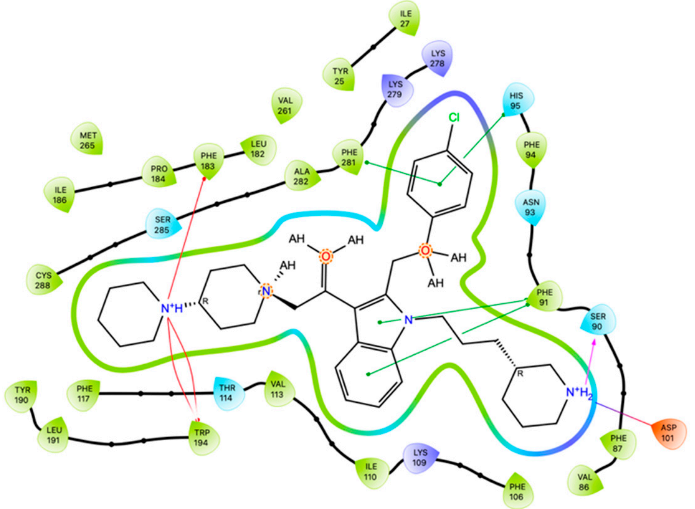	

CCK1 X-ray Structure		Corresponding AF2 model	
Crystal Ligand	PLP-Fitness	Ligand Code	PLP-Fitness
DEVAZEPIDE	78.52	ZINC000036178926	107.575
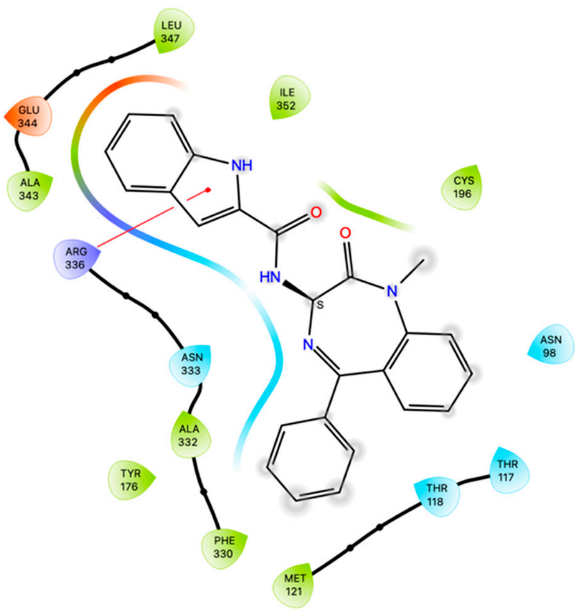		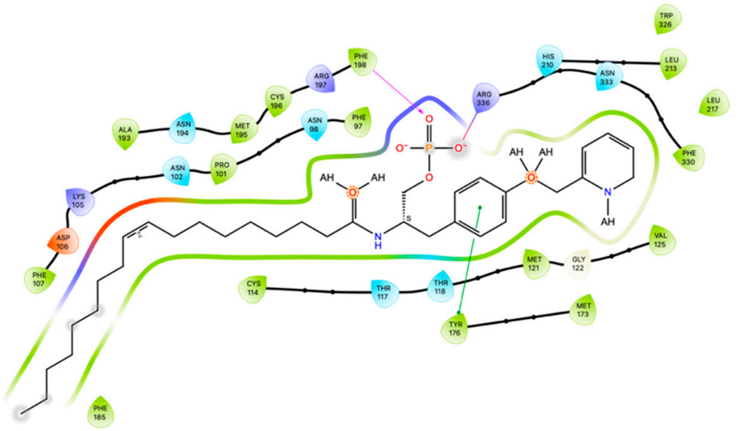	
BLT1 X-ray Structure		Corresponding AF2 model	
Crystal Ligand	PLP-Fitness	Ligand Code	PLP-Fitness
N-(tert-butylsulfonyl)-4-fluoro-2-{(3S,4R)-4-hydroxy-3-[(pyridin-2-yl)methyl]-3,4-dihydro-2H-1-benzopyran-7-yl}benzamide	95.15	ZINC000036178926	109.938
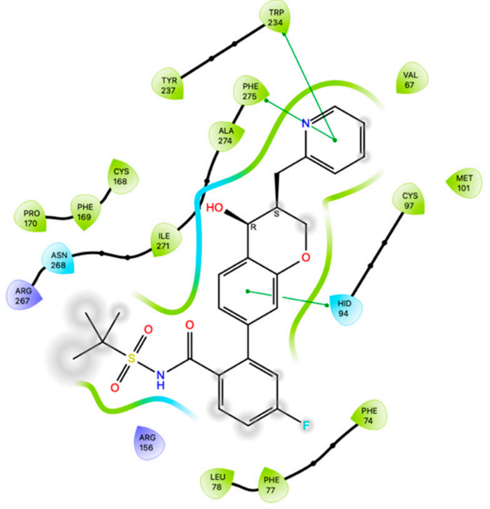		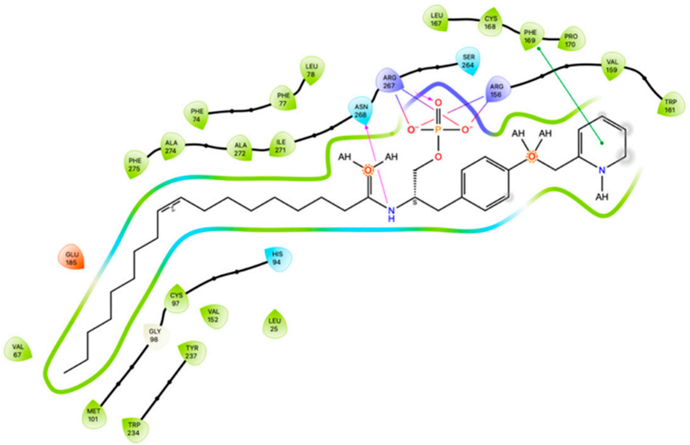	

D2 X-ray Structure		Corresponding AF2 model	
Crystal Ligand	PLP-Fitness	Ligand Code	PLP-Fitness
risperidone	97.06	ZINC000027193871	103.858

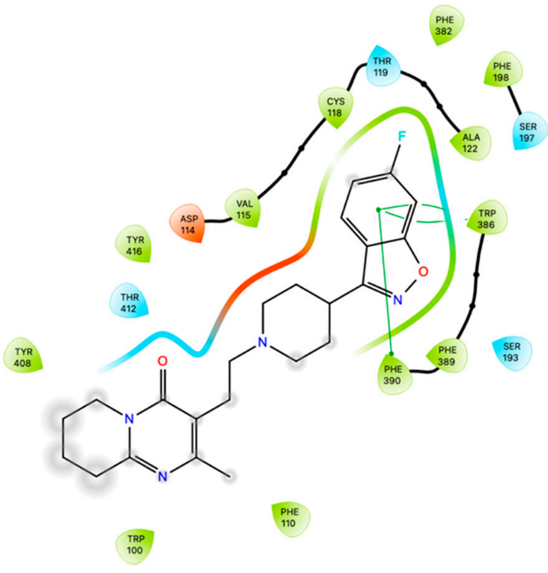

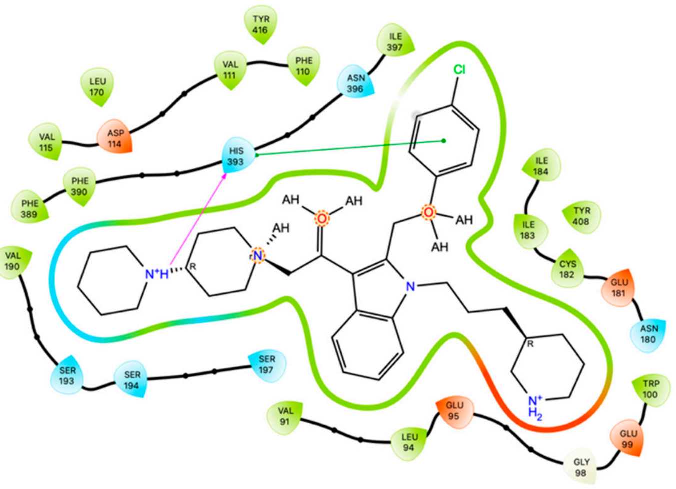

D3 X-ray Structure		Corresponding AF2 model	
Crystal Ligand	PLP-Fitness	Ligand Code	PLP-Fitness
eticlopride	66.29	ZINC000026649493	96.611

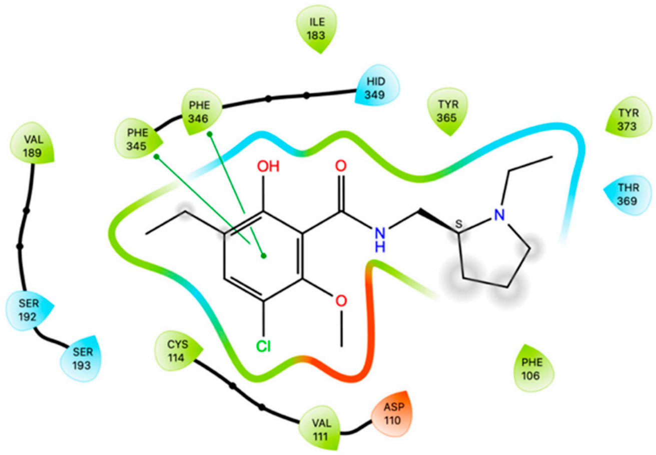

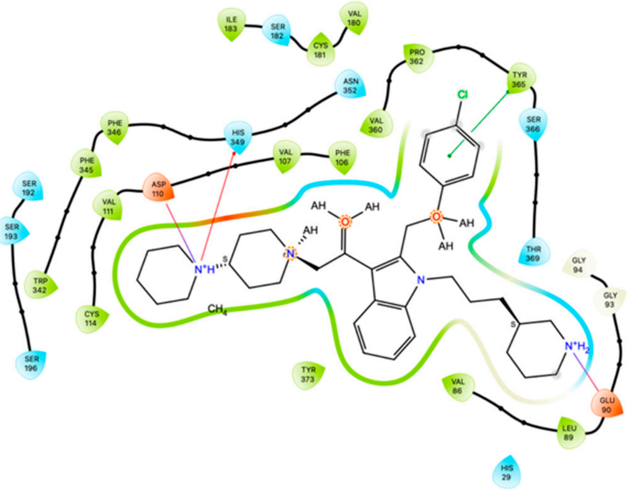

<table>
<tr><td colspan="2">NK1 X-ray Structure</td><td colspan="2">Corresponding AF2 model</td></tr>
<tr><td>Crystal Ligand</td><td>PLP-Fitness</td><td>Ligand Code</td><td>PLP-Fitness</td></tr>
<tr><td>netupitant</td><td>84.99</td><td>ZINC000027193871</td><td>110.332</td></tr>
<tr><td colspan="2">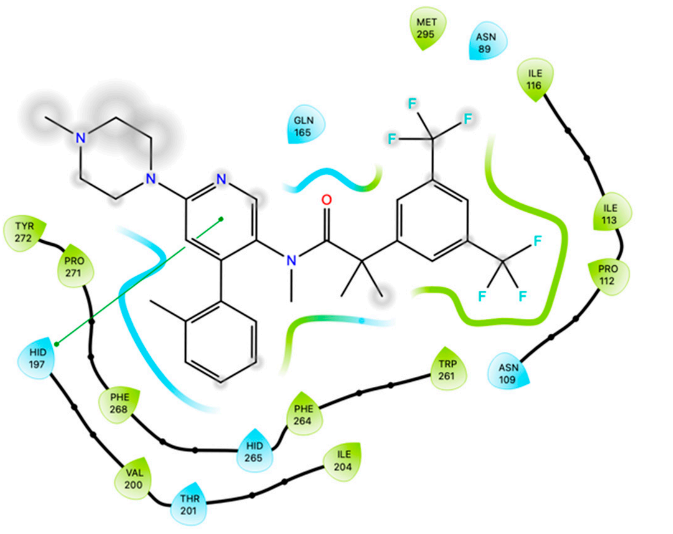
</td><td colspan="2">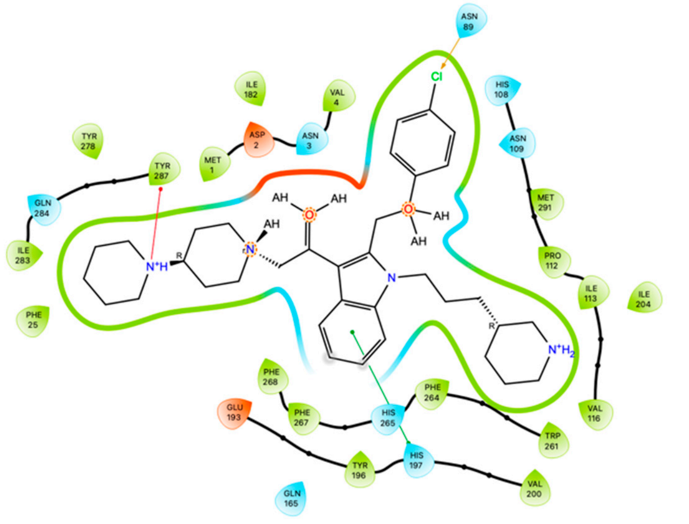
</td></tr>
<tr><td colspan="2">OX2 X-ray Structure</td><td colspan="2">Corresponding AF2 model</td></tr>
<tr><td>Crystal Ligand</td><td>PLP-Fitness</td><td>Ligand Code</td><td>PLP-Fitness</td></tr>
<tr><td>EMPA</td><td>84.43</td><td>ZINC000036178926</td><td>97.458</td></tr>
<tr><td colspan="2">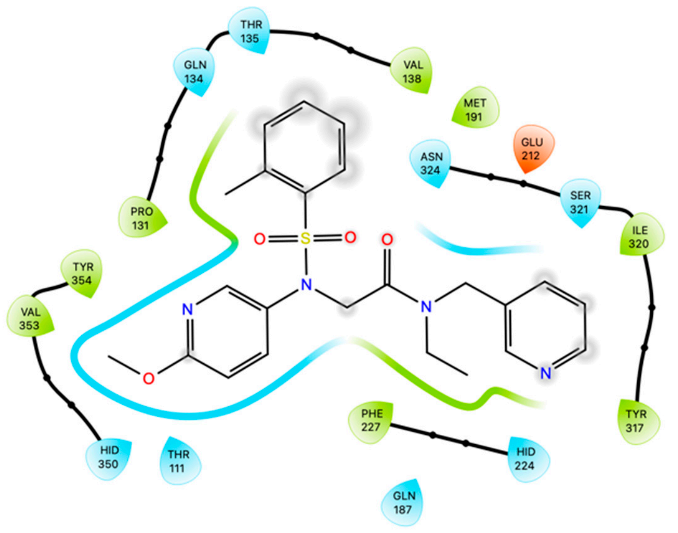
</td><td colspan="2">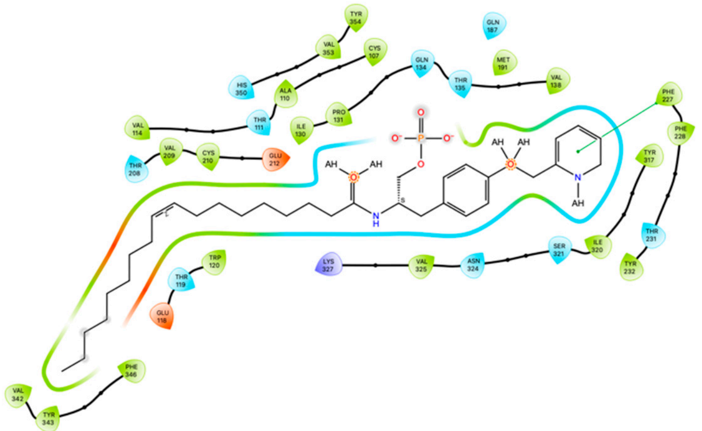</td></tr>
</table>

S1P1 X-ray Structure		Corresponding AF2 model	
Crystal Ligand	PLP-Fitness	Ligand Code	PLP-Fitness
W146	92.10	ZINC000036178926	100.07

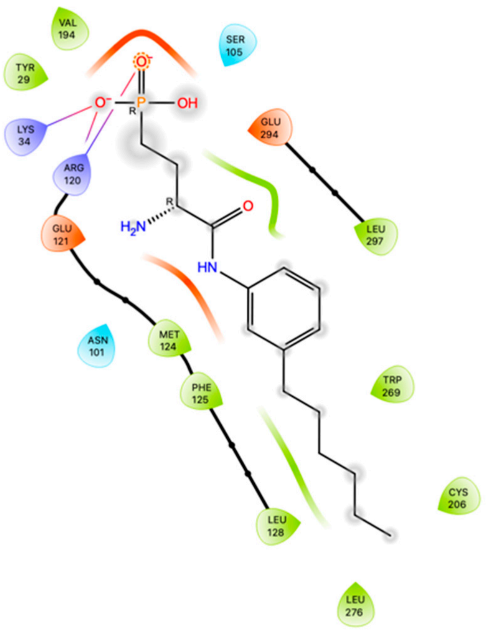

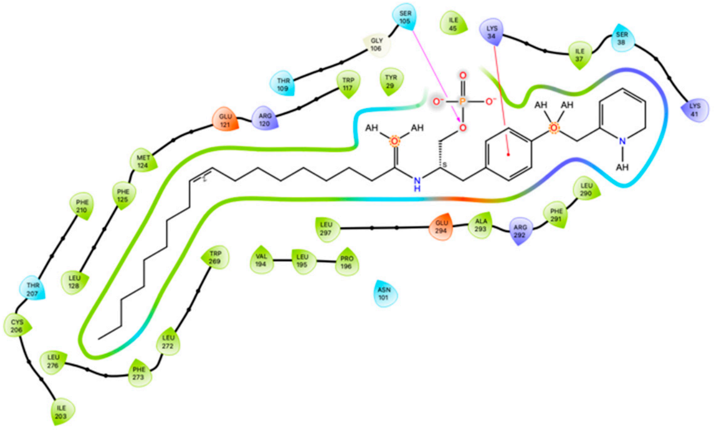

S1P5 X-ray Structure		Corresponding AF2 model	
Crystal Ligand	PLP-Fitness	Ligand Code	PLP-Fitness
4-[6-(2-naphthalen-1-ylethoxy)-2,3,4,5-tetrahydro-1H-3-benzazepin-3-ium-3-yl]butanoic acid	91.40	ZINC000036178926	98.631

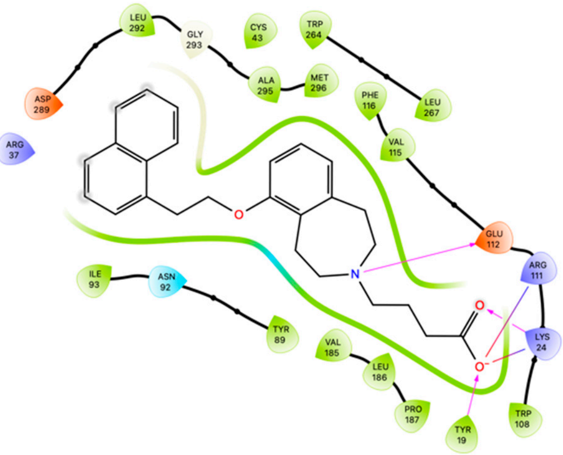

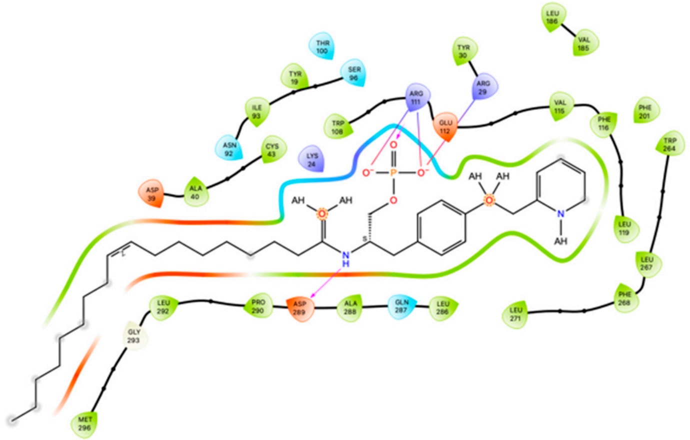

Y2 X-ray Structure		Corresponding AF2 model	
Crystal Ligand	PLP-Fitness	Ligand Code	PLP-Fitness
CHEMBL1823578	101.70	ZINC000026394331	115.938

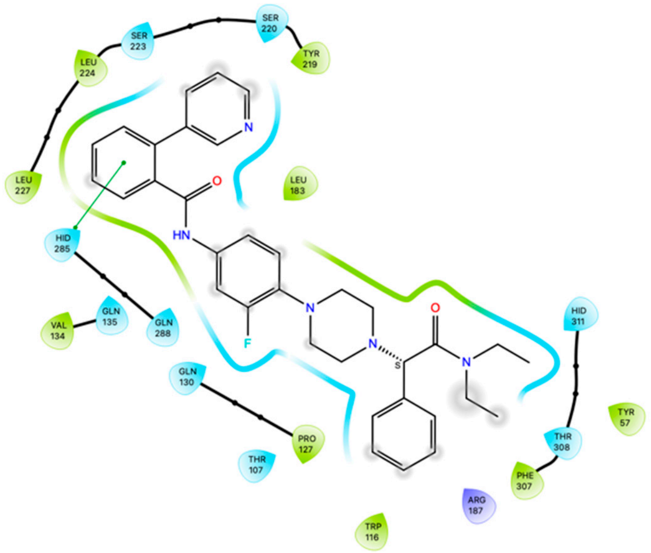

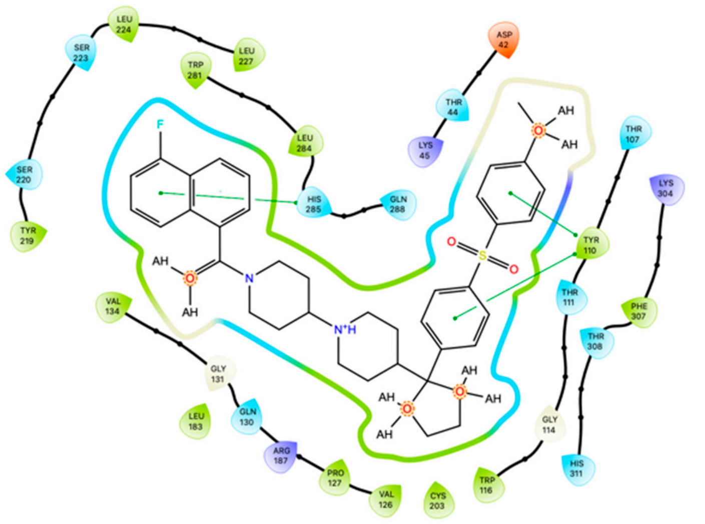

α2A X-ray Structure		Corresponding AF2 model	
Crystal Ligand	PLP-Fitness	Ligand Code	PLP-Fitness
RSC	67.60	ZINC000036178926	83.862

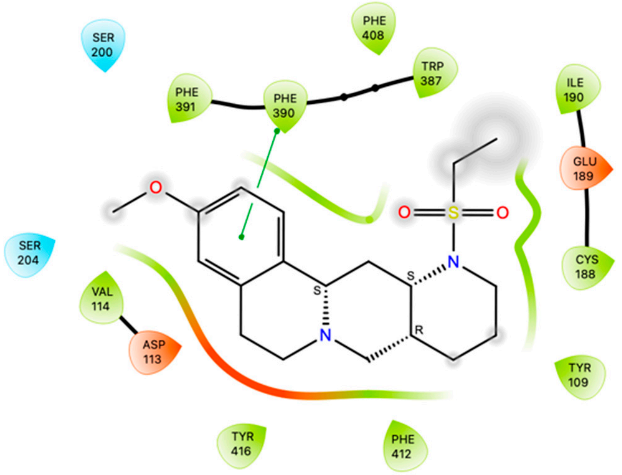

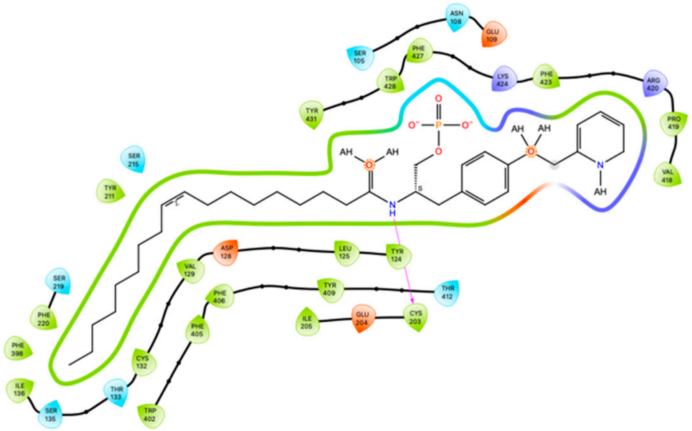

β1 X-ray Structure		Corresponding AF2 model	
Crystal Ligand	PLP-Fitness	Ligand Code	PLP-Fitness
(S)-Cyanopindolol	66.00	ZINC000026384753	104.35
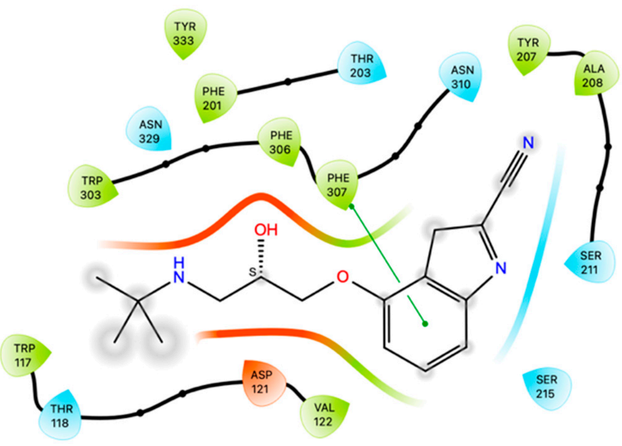		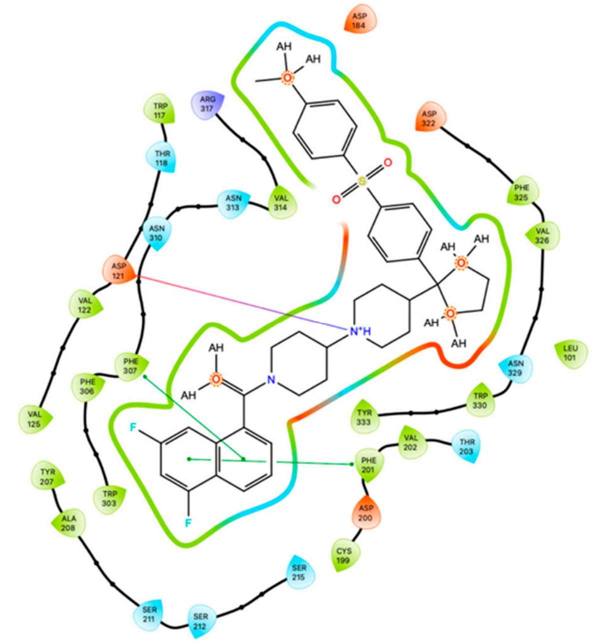	
κ X-ray Structure		Corresponding AF2 model	
Crystal Ligand	PLP-Fitness	Ligand Code	PLP-Fitness
JDTic	85.02	ZINC000013604972	105.952
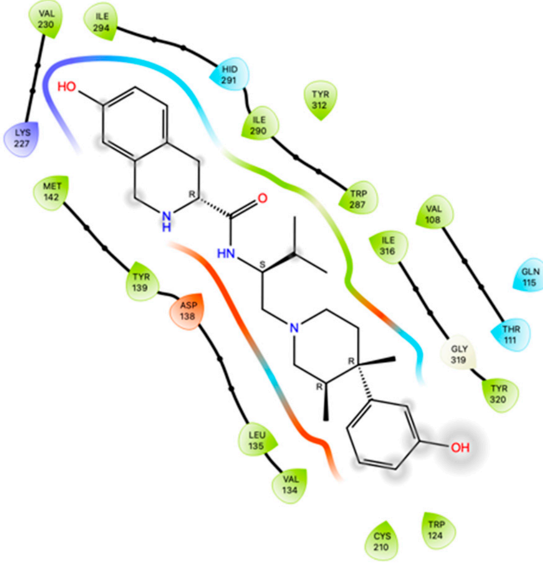		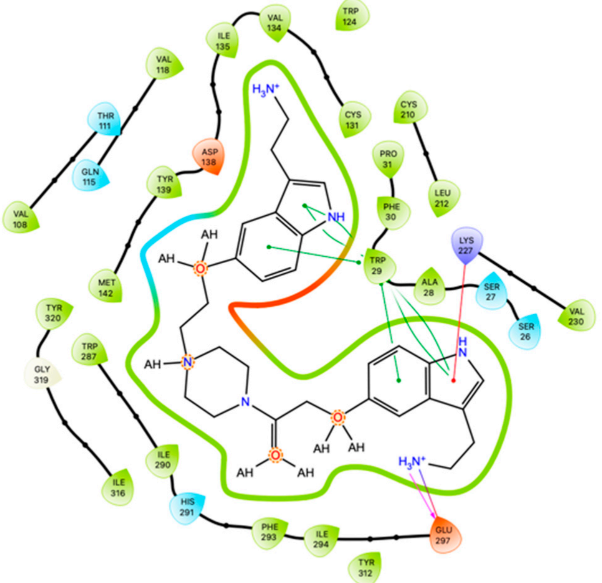	

μ X-ray Structure		Corresponding AF2 model	
Crystal Ligand	PLP-Fitness	Ligand Code	PLP-Fitness
BU72	78.48	ZINC000036178926	105.022
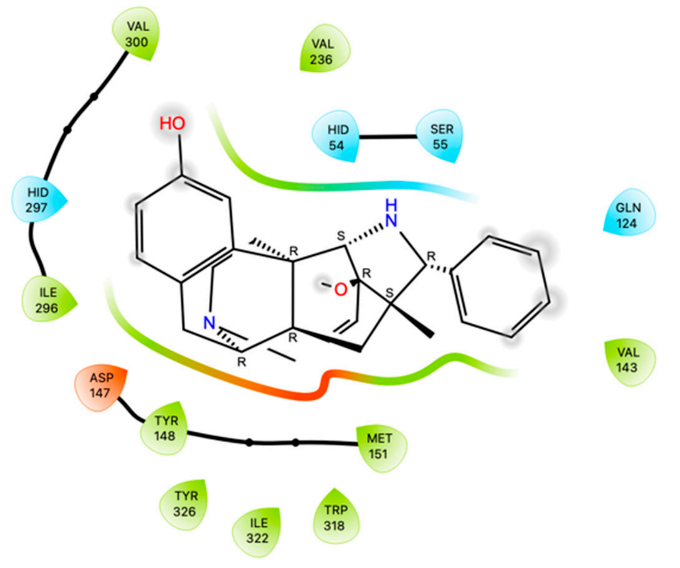		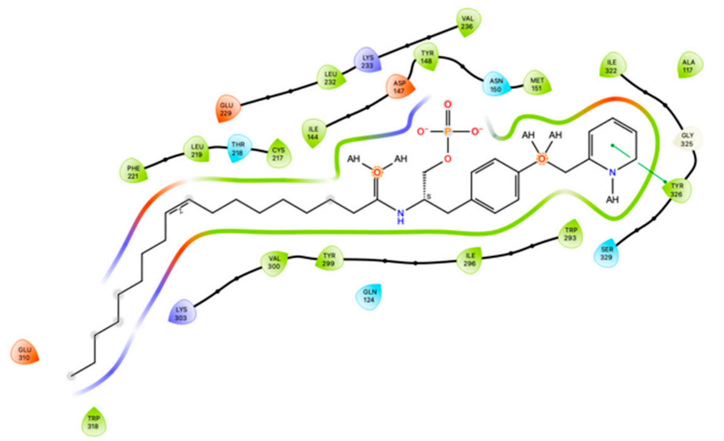	
